# Commentary: Statistical Modeling for the Prediction of Infectious Disease Dissemination With Special Reference to COVID-19 Spread

**DOI:** 10.3389/fpubh.2021.735857

**Published:** 2021-10-15

**Authors:** Hom Nath Dhungana, Saroj Ghimire

**Affiliations:** ^1^School of Mathematical Science, University of Technology Sydney, Sydney, NSW, Australia; ^2^School of Public Health, Torrens University, Sydney, NSW, Australia

**Keywords:** infectious disease modeling, reproduction number, mathematical modeling, transmission rate, effective contact rate

This commentary builds upon the recent study of Subhash Kumar Yadav and Yosuf Akhter entitled “*Statistical Modeling for the Prediction of Infectious Disease Dissemination With Special Reference to COVID-19 Spread*” published in Frontiers in Public health. The study describes the basics of the mathematical epidemiology of infectious diseases. The study attempted to introduce a history of epidemic modeling and pointed out some standard methods of modeling. After careful reading of the study, we identified erroneous definitions in multiple sections other than typos.

In sections “SI and SIS Models” and “SIR and SIRS Models,” the authors define β as an infectious rate and further write β as a chance, which is a misinterpretation of β. Additionally, in section “SI With Vital Dynamics,” the authors again wrongly define β as a rate of infection. Precisely, β is the per capita rate at which two different individuals come in effective contact per unit time (1). It neither refers to a chance or probability of infection nor is an infection rate. For a correct definition of infection rate in detail, please refer to the book (2), page number 127. To understand the basic difference between contact rate and transmission rate, we can use following simple SI model.

Figure 1 represents a simple SI model, where S is the size of susceptible class and I is the size of infected class at time t. At the beginning of infection, the size of susceptible class decreases; therefore, the size of infected class increases. Then, at time *t* + 1, the size of susceptible will be


$$S_{t+1}=S_{t}-\lambda_{t}S_{t}$$

where λ_*t*_ is called the force of infection or the risk of infection at time t and it varies with time. In 1906, W.H. Hamer postulated that the transmission of infection should depend on the number of susceptible individuals, the number of infective individuals, and the contact rate between them. So, the net rate of transmission of infection is proportional to the multiplication of the product of the density of susceptible and the density of infected. This statement has been recognized as a mass action principle in epidemiology and is a base of modern mathematical epidemiology and compartment models of infectious diseases. Therefore, λ_*t*_ = β*I*_*t*_. Authors mistakenly write β as probability of infection or transmission probability as it does not follow the basic probability axioms. β*SI* is called transmission rate or an instantaneous rate from S to I, and it is not a chance or probability.

**Figure 1:**
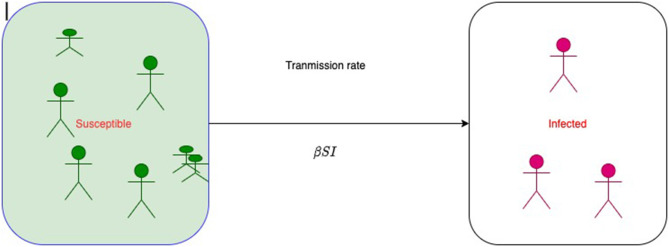
A simple SI model without demography. β is per capita contact rate.

In section, “The Distribution Fitting,” the authors mentioned that the infectious disease mainly depends on two factors, namely, the number of carriers and the time of infections. This line is very vague, especially for readers and definitely, and it needs improvements in terms of context writing. The possible context might be the infectious disease transmissibility, where the contagiousness of a pathogen depends on the chance of infection (infectivity), duration of infectiousness, and effective contact rates. Therefore, the authors should explain the context and the frame of the sentence with their biological interpretations.

In the section of “The Basic Reproduction Number,” the authors defined the basic reproduction number and the factors it depends on. Further, the authors wrote that *R*_0_ is a unit-free quantity (without dimension of measurement), which is correct. However, in the following line, the authors used the word reproductive rate, which is inconsistent and confusing. Many prominent researchers in the epidemic modeling have suggested that the terms “reproductive rate” or “reproduction rate” are wrong because the estimation of *R*_0_ does not involve time as a function of it. Moreover, in the compartmental model, the reproduction number *R*_0_ can be calculated by using next-generation matrix method (NGMM) (3).

We define a matrix G that comprises two matrices F and *V*^−1^ as follows,


$$F=\left[\frac{\partial F_{i}\left(x_{0}\right)}{\partial x_{j}}\right]$$

and


$$V=\left[\frac{\partial V_{i}\left(x_{0}\right)}{\partial x_{j}}\right]$$

where *F*_*i*_ is the new infections, while the *V*_*i*_ is the transfers of infections from one compartment to another. *x*_0_ is the disease-free equilibrium state. The dominant eigenvalue of G = *FV*^−1^ is *R*_0_. In NGMM, the dominant eigenvalue does not represent any rate. However, it is very common to see such key-words as rates in routine publications and in even some books. If *R*_0_ was a rate involving time, the measure would provide information about how fast an epidemic will spread through a population. More details, on how *R*_0_ is not a rate but is a pure number, can be read in some highly influencing studies (4, 5). There are numerous inconsistencies in the name of reproduction number (4); therefore, we suggest authors at least follow a specific name (either rate or number) consistently throughout the study to avoid confusions for readers.

In section, “SIR and SIRS Models,” the authors mentioned “The SIRS model ξ represents the transmission rate from recovered to susceptible state because of decay in immunity” (page number 9), where ξ is an instantaneous rate from recovery class to susceptible class, and this line needs to be corrected, because ξ is a model parameter not the model.

In the section, “Further Suggestions and Future Prospectives,” the authors put a couple of suggestions, especially on sample size issues in modeling. It is vital in inferential statistics to incorporate the sample size effect on parameter estimation (sample size must be appropriately calculated). However, this compulsion can be avoided for the epidemic curve-based models using serial interval data and disease incidence. This means, to model short-term infections (SARS, MERS, and Influenza), some preamble methodologies do not have any assumptions about the sample size (6–8). To estimate the reproduction number of such short-term infection using epidemic curve-based models, we do not make any assumptions on sample size but it requires some assumptions on growth rate (6, 7, 9, 10). For example, if a school children get infected by COVID-19 and infects some other students of the class within few days and infection grows, we can estimate *R*0 and other parameters (β, γ) by doing model fitting even for small number of time series data without violating any model assumptions. Therefore, authors need to present specific models/methods that are significantly affected by sample size before generalizing the recommendations.

## Author Contributions

HD drafted the manuscript and SG approved the final version the manuscript. Both authors contributed to the article and approved the submitted version.

## Conflict of Interest

The authors declare that the research was conducted in the absence of any commercial or financial relationships that could be construed as a potential conflict of interest.

## Publisher's Note

All claims expressed in this article are solely those of the authors and do not necessarily represent those of their affiliated organizations, or those of the publisher, the editors and the reviewers. Any product that may be evaluated in this article, or claim that may be made by its manufacturer, is not guaranteed or endorsed by the publisher.
